# Intersections between proteostasis and immunity: insights from *Caenorhabditis elegans*

**DOI:** 10.1242/dmm.052534

**Published:** 2026-02-04

**Authors:** Emily R. Troemel, Patricija van Oosten-Hawle, Michalis Barkoulas

**Affiliations:** ^1^ Department of Cell and Developmental Biology, School of Biological Sciences, University of California, San Diego, La Jolla, CA 92093-0349, USA; ^2^ Department of Biological Sciences, University of North Carolina at Charlotte, Charlotte, NC 28223, USA; ^3^ Department of Life Sciences, Imperial College, London SW7 2AZ, UK

**Keywords:** Proteostasis, Innate immunity, Pathogen, *C. elegans*, Infection

## Abstract

Cells must properly synthesize, fold and degrade proteins to maintain protein homeostasis, or proteostasis. Studies in the model nematode host *Caenorhabditis elegans* have illuminated different ways in which proteostasis intersects with immune responses against pathogen infection, which is the focus of this Review. For example, pathogens often interfere with host proteostasis pathways to survive and replicate. Hosts, in turn, can sense these perturbations and then trigger immune responses, creating additional burdens on proteostasis. This Review is organized by the cellular compartments in which proteostasis pathways are activated, starting with the cytosolic processes of protein synthesis, folding, degradation and the ubiquitin–proteasome system. Next, we cover autophagy and lysosome-related processes, followed by pathways triggered in the endoplasmic reticulum and mitochondria. We discuss infections in *C. elegans* by bacteria, viruses, microsporidia and oomycetes; all of these pathogen types infect humans. We provide examples of how findings in *C. elegans* relate to mammals, noting how the coordination of proteostasis and immunity can be conserved across species. We emphasize a recurring theme in *C. elegans* that impairment of one proteostasis pathway can lead to compensatory activation of another pathway, ultimately providing a health benefit to the host, highlighting organismal resilience.

## Teaser

**Summary:** Discoveries from the nematode *C. elegans* have provided insight into the many connections between immune responses and proteostasis, with examples of conservation across species.

## Introduction: how pathogen infection can perturb host proteostasis

**Fig. 1.**
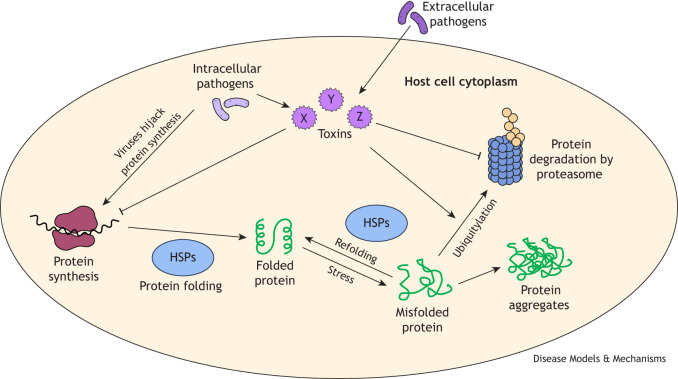
**General steps of host proteostasis and impact of pathogens.** Shades of purple indicate pathogens or pathogen-derived toxins (X, Y, Z). Green represents proteins in various states of synthesis, folding, misfolding or aggregation. Blue represents proteostasis machinery. HSP, heat shock protein.

Homeostasis is the process by which a balance of internal functions is maintained, despite changing external conditions. One of the most important systems requiring such regulation is the set of proteins that executes cellular processes and includes enzymes, transmembrane channels, transcription factors, signaling proteins and a myriad of other protein classes. In 2008, a group of researchers including the *Caenorhabditis elegans* researchers Rick Morimoto and Andy Dillin coined the term ‘proteostasis’ to refer to protein homeostasis. Since then, proteostasis has become a highly significant area of study, which has focused on how the processes of protein synthesis, folding and degradation are tightly monitored and regulated in the face of intrinsic or external challenges (Fig. 1, Table 1) (Balch et al., 2008). These processes are highly conserved across species (Table 1) and offer insight into virtually all areas of human health, especially neurodegenerative diseases and other aging-related diseases such as Alzheimer's disease (AD), in which perturbed proteostasis can be manifested with misfolded proteins and protein aggregates as a hallmark of this growing health problem (Marasco, 2020; Ruano, 2021; Butterfield and Boyd-Kimball, 2018).

**Table 1.** Conservation of *Caenorhabditis elegans* proteostasis pathways discussed in this Review

Proteostasis pathway	*C. elegans*	*Homo sapiens*	References
Blockade of translation initiation (integrated stress response)	EIF-2A kinases GCN-2 and PEK-1; transcription factor ATF-4	EIF2A kinases GCN2, PERK, HRI and PKR; transcription factor ATF4	Costa-Mattioli and Walter, 2020; Ma et al., 2023
Heat shock response	Transcription factor HSF-1 induces HSPs	Transcription factor HSF1 induces HSPs	Kyriakou et al., 2022; Fujimoto and Nakai, 2010; Lindquist, 1986; Cotto and Morimoto, 1999
Skp-cullin F-box ubiquitin ligases	FBXA-75,158/CUL-6/SKR-3,4,5/RCS-1 induce thermotolerance	CUL1/SKP1 are homologs; unclear relationships for RCS-1 and FBXA-75,158*; unknown role in thermotolerance	*For more information on homolog/ortholog relationships, see Lažetić and Troemel, 2020
Proteasome	20S core particle with 14 subunits (PAS-1-7, PBS-1-7); 19S regulatory particle with 19 subunits (RPT-1-6, RPN-1-3, RPN-5-11)	20S core particle with 14 subunits (PSMA1-7, PSMB1-7); 19S regulatory particle with 19 subunits (PSMC1-6, PSMD1-14)*	*For more information on homolog/ortholog relationships, see Papaevgeniou and Chondrogianni, 2014; Finley and Prado, 2020
Proteasome inhibition response	Controlled by SKN-1A induction of proteasome subunits	Controlled by NRF1 induction of proteasome subunits	Lehrbach, 2022
Endoplasmic reticulum unfolded protein response	Three sensors: IRE-1/XBP-1, PEK-1 and ATF6	Three sensors: IRE1/XBP1, PERK and ATF6	Lee, 2021, Frakes and Dillin, 2017
Autophagy	UNC-51, ATG-13, BEC-1, VPS-34, LGG-1/2, SQST-1	ULK1, ATG13, BECN1, PIK3C3, LC3, SQSTM1*	*For more information on homolog/ortholog relationships, see Kuo et al., 2018; Lange et al., 2025
Mitochondria unfolded protein response	Transcription factor ATFS-1 redirected from mitochondria to nucleus to induce defense genes	Transcription factor ATF5 redirected from mitochondria to nucleus to induce defense genes	Kim et al., 2024; Naresh and Haynes, 2019

One of the major insults to organismal proteostasis is pathogen infection. The tissues, subcellular compartments and protein systems impacted by infection will depend on the type of pathogen, which can broadly be placed into three groups: (1) obligate intracellular pathogens, such as viruses, which complete their entire replicative life cycle inside cells; (2) facultative intracellular pathogens that can replicate either inside or outside the cell; and (3) extracellular pathogens. Notably, viruses usually lack protein synthesis and folding machinery and, thus, often hijack host pathways (Rozman et al., 2023; Aviner and Frydman, 2020) (Fig. 1). Pathogens deploy toxins that disrupt host proteostasis, such as by blocking host mRNA translation factors or hijacking the ubiquitin–proteasome system to degrade specific host proteins (Fig. 1) (Popoff, 2024; Bullones-Bolaños et al., 2022; Sun et al., 2023). Obligate and facultative pathogens can deliver these toxins across host intracellular membranes using specialized secretion systems, whereas extracellular pathogens can hijack host endocytosis to transport toxins into host cells. In addition to direct delivery, intracellular pathogens can impact host proteostasis simply through their replication, which reduces the amount of intracellular space for host factors, causing crowding in the cell (Balla et al., 2016). Beyond pathogen-derived impacts, the host immune response itself can cause proteotoxic stress – for example, through the upregulated production of secreted anti-microbials and cytokines, which must pass through the secretory system, requiring activation of the endoplasmic reticulum unfolded protein response (ER-UPR) (Richardson et al., 2010). Thus, host proteostasis pathways intersect with infection and immunity in several ways.

This Review provides examples from the model organism nematode *C. elegans* that illustrate how pathogens can impact host proteostasis, how the host senses and responds to these effects, and how these responses can improve defense against infection and help restore host proteostasis. The motivation for covering this topic now is the growing list of examples described below, for which there are surprising benefits in the context of immunity and aging resulting from proteotoxic stress that might be expected to cause harm. Here, studies in *C. elegans* have helped highlight how proteostasis pathways are not just generalized stress responses. Instead, they have specific and varying roles depending on the pathogen type infecting the host, the types of toxins being delivered and the affected subcellular region. These interactions often happen in epithelial cells, which are among the first cell types to be infected, yet they remain less well studied than professional immune cells (Sharma et al., 2020). Thus, the interplay between proteostasis and immunity is particularly relevant to many infections in humans that occur in epithelial cells of the intestine and skin, as well as inflammatory conditions in these tissues, such as inflammatory bowel diseases (Hooper, 2015; Liebing et al., 2025).

## *C. elegans* as a model for infection

**Fig. 2.**
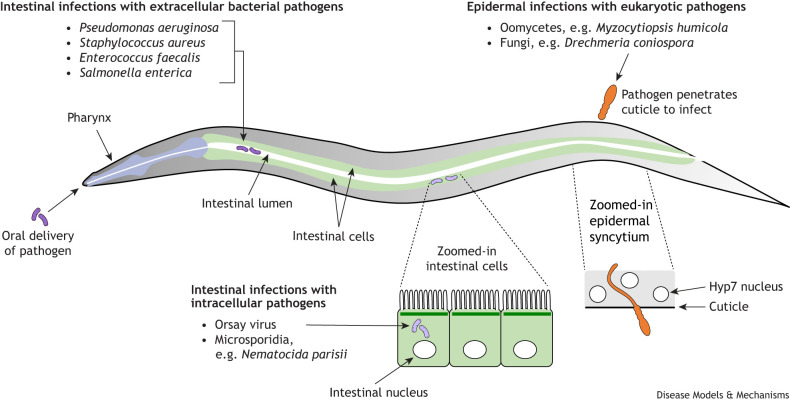
**How pathogens discussed in this Review are delivered to *Caenorhabditis elegans* and what tissues they infect.** *C. elegans* can be infected through two main routes: oral delivery and cuticle penetration. Oral delivery mostly leads to intestinal infections (in purple), which can be either intracellular (Orsay virus, microsporidia) or extracellular (bacteria). Pathogens (depicted in orange) can also penetrate the cuticle to infect, such as in the case of fungi and oomycetes. Zoomed insets show detail of intestinal cells with nuclei and the epidermal syncytium with hyp7 nuclei.

*C. elegans* has a simple body plan that contains many of the major tissue types found in humans – including neurons, epidermis (also called hypodermis in *C. elegans*), intestine and muscle – but with remarkably reduced complexity of just 959 non-renewing, terminally differentiated somatic cells in the adult hermaphrodite (Fig. 2) (Cheddadi et al., 2024; Corsi et al., 2015). This simplicity and the proteostasis signaling pathways conserved across species (Table 1) have made *C. elegans* an excellent system for dissecting interactions between these pathways and immunity (Miles et al., 2019). Another advantage of *C. elegans* is its transparency, which facilitates microscopy in a whole-animal context, allowing measures of proteotoxic stress, such as build-up of aggregated proteins, and measures of pathogen replication and pathogen load during pathogen infection. *C. elegans* is widely used as a model organism across diverse fields, including aging, and its small size, fast generation time and short lifespan are some of the features that have enabled ground-breaking studies, such as the discovery that lifespan can be genetically controlled by insulin-like signaling (Kenyon et al., 1993). Interestingly, such pathways often regulate multiple physiological processes; for example, the insulin-like signaling pathway regulates lifespan, pathogen resistance and resistance to proteotoxic stress in *C. elegans* (Biglou et al., 2021). The many strengths of *C. elegans* as a tractable laboratory system have led to Nobel-prize-winning discoveries over the years, including the discovery of CED-3/caspase and other apoptosis genes (Ellis and Horvitz, 1986) and, more recently, the discovery of RNA interference (Fire et al., 1998) and microRNAs (Lee et al., 1993; Reinhart et al., 2000; Pasquinelli et al., 2000).

Studies using *C. elegans* for immunity research began around 1999, with reports of *Pseudomonas aeruginosa* causing lethal intestinal infections (Mahajan-Miklos et al., 1999; Tan et al., 1999; Darby et al., 1999). Subsequently, several other pathogens were discovered or introduced in this model, with most pathogens studied in *C. elegans* targeting either the intestine or the epidermis (Martineau et al., 2021; Tran and Luallen, 2024) (Table 2, Fig. 2). The tissue targeted usually depends on the pathogen's mode of entry into the host. Intestinal infections occur via ingestion, whereas most epidermal infections result from penetration of the protective extracellular layer known as the cuticle (Fig. 2). Other clinically relevant bacterial species such as *Staphylococcus aureus* and *Enterococcus faecalis* have been useful pathogens to study *C. elegans* immune responses, yet *P. aeruginosa* strain PA14 remains the most commonly used pathogen in *C. elegans* immunity studies (Hajdú et al., 2024). In humans, *P. aeruginosa* typically causes infections when tissue defenses are compromised – for example, in burn patients, from whom the PA14 strain was originally isolated (Schroth et al., 2018). In *C. elegans*, PA14 causes an intestinal infection leading to death in a couple of days, beginning as an extracellular pathogen that invades the intestinal cells as tissue damage progresses (Irazoqui et al., 2010a). The findings on *P. aeruginosa* described in this Review are from the PA14 strain, which will be referred to as *P. aeruginosa* going forward. This strain has been particularly useful because of its high level of pathogenicity in *C. elegans* and the many well-characterized virulence factors that are important in humans and in *C. elegans* (Table 2) (Hajdú et al., 2024).

**Table 2.** Pathogens of *C. elegans* discussed in this Review

Species name, type and isolation	Infection mode	Assay conditions	Features of infection in *C. elegans*	References
*Pseudomonas aeruginosa*; Gram-negative bacteria; strain PA14 isolated from human burn patient	Predominantly extracellular intestinal	Slow-killing agar conditions: pathogen replaces normal food source and must be alive. Fast-killing agar conditions: involves toxins and does not require pathogen to be alive. Liquid-killing conditions: disrupts iron homeostasis.	PA14 is the most commonly studied pathogen in *C. elegans.* Some virulence factors important for pathogenicity in mammals are also important in *C. elegans*, including small molecules such as phenazines and pyoverdine, and proteins such as Exotoxin A and the GacA/GacS response regulator. Begins extracellularly and becomes intracellular in late-stage infection in the slow-killing model.	Mahajan-Miklos et al., 1999; Tan et al., 1999; Irazoqui et al., 2010a; Kirienko et al., 2013; McEwan et al., 2012; Dunbar et al., 2012; Kang et al., 2018
*Salmonella enterica*; Gram-negative bacteria; strain SL1344 isolated from cattle with salmonellosis	Extracellular intestinal	Pathogen replaces normal food source.	Causes persistent infection in intestinal lumen (i.e. infection is maintained after transferring animals off lawn of pathogen onto *E. coli* lawn); virulence factors important for pathogenicity in mammals are also important in *C. elegans*, including PhoP/PhoQ. Mixed reports on whether it becomes intracellular inside *C. elegans*; is a facultative intracellular pathogen in mammals.	Aballay et al., 2000; Labrousse et al., 2000; Jia et al., 2009; Curt et al., 2014
*Staphylococcus aureus*; Gram-positive bacteria; strain NCTC8325 isolated from human patient with sepsis	Extracellular intestinal	Pathogen replaces normal food source; grow on rich media.	Causes enterocyte effacement (loss of intestinal microvilli).	Garsin et al., 2001; Irazoqui et al., 2010a
*Enterococcus faecalis*; Gram-positive bacteria; strain OG1RF is a derivative of a human isolated strain	Extracellular intestinal	Pathogen replaces normal food source; grow on rich media.	Causes persistent infection in intestinal lumen; virulence factors important for pathogenicity in humans also important in *C. elegans*, including Fsr genes, a putative quorum-sensing system and cytolysin.	Garsin et al., 2001
Enterohemorrhagic *Escherichia coli*; Gram-negative bacteria; strain O157:H7 isolated from human	Extracellular intestinal	Pathogen replaces normal food source.	Causes paralysis and killing; requires tryptophanase and shiga-like toxin 1 for pathogenicity; causes attaching and effacing lesions, similarly to infections in mammals.	Anyanful et al., 2005; Kenney et al., 2005; Chou et al., 2013; Bommarius et al., 2013; Youn et al., 2013
*Bacillus thuringiensis* Cry5B; Gram-positive bacteria	Extracellular intestinal	Fed on *E. coli* expressing Cry5B toxin.	Cry5B pore-forming toxin causes pathogenicity; studies in *C. elegans* provided basis for studies to use Cry5B as a therapeutic to treat parasitic nematode infections in humans.	Griffitts et al., 2001; Kho et al., 2011; Hu et al., 2012
*Myzocytiopsis humicola*; oomycete (eukaryote); wild-caught *C. elegans*	Extracellular epidermal	Pathogen added to food.	One of several oomycete species isolated from *C. elegans* in the wild. Penetrates the cuticle and causes excessive tissue degradation. Oomycetes are generally considered plant pathogens but can also infect humans using the life cycle as in *C. elegans* infections.	Osman et al., 2018; Grover et al., 2021
*Nematocida parisii*; microsporidia (fungus); wild-caught *C. elegans*	Obligate intracellular intestinal	Pathogen added to food.	One of many species of microsporidia found naturally infecting *C. elegans* in the wild. Microsporidia phylum has pathogens that infect humans. All microsporidia use polar tube infection apparatus to invade cells. Once inside cells, *N. parisii* replicates in contact with cytosol, similar to the human-infecting species *Enterocytozoon bieneusi*, which is unculturable. *N. parisii* causes fusion of intestinal cells to facilitate spread and hijacks host exocytosis for non-lytic exit from intestinal cells. Horizontally but not vertically transmitted.	Troemel et al., 2008; Zhang et al., 2016; Szumowski et al., 2014; Balla et al., 2016
Orsay virus; positive sense single-stranded RNA virus; wild-caught *C. elegans*	Obligate intracellular intestinal	Pathogen added to food.	Only virus so far shown to undergo entire infection cycle inside *C. elegans*. Causes disruption of intestinal cell integrity. Mostly restricted to the intestine; horizontally but not vertically transmitted.	Félix et al., 2011; Fujii et al., 2025
			For more information on other *C. elegans* pathogens, e.g. *Drechmeria coniospora*, see reviews specifically on *C. elegans* pathogens.	Marsh and May, 2012; Schulenburg and Félix, 2017

A growing number of pathogens and associated microbes have been isolated from wild-caught *C. elegans* (Table 2, Fig. 2), including environmentally isolated *Pseudomonas* and other bacterial species (Schulenburg and Félix, 2017). Beyond the bacterial domain, microsporidia (obligate intracellular fungi) are common pathogens isolated from *C. elegans* in the wild, with the species *Nematocida parisii* being first described in 2008 and the most common. *N. parisii* infects the intestine, completing its entire replicative life cycle in this tissue (Troemel et al., 2008; Zhang et al., 2016; Tecle and Troemel, 2022; Gang and Lažetić, 2024). In 2011, a naturally occurring single-stranded positive-sense RNA virus called the Orsay virus was described and, to date, remains the only known viral species that can complete its replication cycle in *C. elegans* (Félix et al., 2011; Wu et al., 2025; Félix and Wang, 2019). More recently, wild-caught *C. elegans* have been found infected with oomycetes (eukaryotic pathogens that are genetically distinct from fungi but resemble them morphologically), which enter through the cuticle (Osman et al., 2018; Grover et al., 2021; Grover and Barkoulas, 2021). All of these natural pathogens are being studied in *C. elegans* immunity research.

Similarly to in aging and stress response studies, a common assay for *C. elegans* immunity has been assessing survival upon infection (Kwon and Lee, 2025). In the case of *C. elegans*, pathogens are generally delivered not through injection (a common practice in other host models), but by transferring organisms from their normal food source of nonpathogenic *Escherichia coli* onto a lawn containing the pathogen as its food source (Foster et al., 2020). The exact design of the survival assay depends on the type of pathogen studied (Table 2). For example, the size of the lawn can be adjusted to cover the entire plate, eliminating the possibility that behavioral avoidance influences the outcome of an infection. Instead of transferring onto an entire lawn of pathogen that also serves as a food source, the pathogen can instead be delivered together with the food source (*E. coli*) in various forms, such as fungal/microsporidian spores and virions from laboratory preparations (Batachari et al., 2024b; Bakowski et al., 2014), or infected animals releasing oomycete zoospores co-cultured on the *E. coli* lawn together with uninfected animals (Osman et al., 2018). To show specificity in survival assays, it is essential to control for animal fitness and processes such as feeding, defecation, reproduction and egg-laying, which may vary across different strain backgrounds and indirectly affect the rate at which animals die upon infection.

There are two distinct defense strategies for any host: (1) defense against infection conferred by lowering the pathogen load, which is defined as increasing ‘pathogen resistance’; and (2) defense conferred by improving health outcomes, which is defined as increasing ‘pathogen tolerance’ or ‘disease tolerance’ (McCarville and Ayres, 2018). Although some *C. elegans* studies incorporate pathogen load assays, which provide a critical early read-out to distinguish between the two strategies, the standard survival assays mentioned above are unable to do so. Therefore, using appropriate read-outs to inform on the defense strategy is important to better understand potential mechanisms at play.

The immune system of *C. elegans* is compared with the immune systems of humans and bacteria in Table 3. So far, all *C. elegans* immune responses described in the literature are mediated by ‘non-professional’ immune cells. Although *C. elegans* has scavenging cells called coelomocytes (Fares and Greenwald, 2001), these cells do not have a demonstrated role in immunity. Thus, it appears that *C. elegans* does not have scavenging ‘professional’ immune cells analogous to macrophages in mammals or hemocytes in *Drosophila melanogaster* (Meister and Lagueux, 2003). The non-professional immune cells in *C. elegans* include the 20 terminally differentiated epithelial cells that comprise the intestine and the main epidermal syncytium (hyp7). Another non-professional immune cell type is neurons, which play a role in sensing infection (Fasseas et al., 2021; Meisel et al., 2014; Liu and Sun, 2021) and transmitting this information to the tissues in which infection commonly occurs.

**Table 3.** Comparison of *C. elegans* immune system to that of other organisms

	*C. elegans*	*Homo sapiens*	Bacteria	References
**Specialized immune cell types**				
Professional adaptive immune cells	Absent	T cells, B cells	Absent (single-celled organisms)	
Professional innate immune cells	Absent (has coelomycetes; no known immune role)	Macrophages, neutrophils	Absent (single-celled organisms)	Sato et al., 2014
**Pattern recognition receptors and other receptors**				
Toll-like receptors	Single homolog TOL-1 does not function as receptor in classic sense	TLR1-10	No clear orthologs	Vijay, 2018; Tse-Kang et al., 2025; Kanzok et al., 2004
RIG-I-like receptors	DRH-1, DRH-2 (potential pseudogene), DRH-3	RIG-I, MDA5	No clear orthologs	Zou et al., 2009
cGAS/STING	No clear orthologs	Present	Present	Rousset, 2023; Patel et al., 2023
NLR proteins	No clear orthologs	22 NLR protein-encoding genes; several act as pathogen sensors	Widespread in bacteria and detect phage proteins to activate defense	Rousset, 2023; Chou et al., 2023
C-type lectin receptors	CLEC-27/CLEC-35; CLEC-26/CLEC-36 detect oomycete infection (molecule unknown)	Dectin-1 recognizes beta-glucans from fungi; dectin-2 recognizes alpha-mannans from fungi	No clear orthologs	Malamud and Brown, 2024; Liu et al., 2024
Nuclear hormone receptors	NHR-86 recognizes phenazine from pathogen *P. aeruginosa*	HNF4 – associated with chronic inflammatory state but no known role in pathogen recognition	No clear orthologs	Rua and Pujol, 2023; Vemuri et al., 2023
**Defense pathways/strategies**				
Apoptosis/cell death	Has developmental caspase-based apoptosis, but no evidence for caspase role in immunity occurring through apoptosis of infected cells	Has caspase-based apoptosis, pyroptosis and many other cell death pathways that kill infected cells and release cytokines	Abortive infection-based cell death of infected cells as anti-phage defense	Bergmann, 2025, Horowitz and Shaham, 2024; Rousset and Sorek, 2023; Kayagaki et al., 2024
TIR-1	TIR-1 catalyzes NAD^+^ and acts upstream of p38 MAPK to promote immunity	SARM1 catalyzes NAD^+^ to activate neuronal cell death	TIR homologs catalyze NAD^+^ to generate cyclic di-nucleotide signaling and cell death	Wani and Pukkila-Worley, 2025
p38 MAPK signaling	NSY-1/SEK-1/PMK-1 promote immunity against many pathogens; upstream of transcription factors ATF-7 and SKN-1	Has orthologs for p38 MAP kinases (MAPKs) and MAPK kinases (MAPKKs) and MAPKK kinases (MAP3Ks); involved in many stress responses	No clear orthologs	Tse-Kang et al., 2025
Sensing of translation elongation blockade	bZIP transcription factors ZIP-2/CEBP-2	Has bZIP transcription factors but no clear orthologs	No clear bZIP transcription factors	Kleino and Silverman, 2012; Tse-Kang et al., 2025
NFκB	No clear orthologs	Major transcription factor acting downstream of cytokine receptors and pattern recognition receptors	No clear orthologs	Irazoqui et al., 2010b
ROS generation by NOX/DUOX family	Two NOX enzymes: Ce-Duox2 (no known role) and Ce-Duox1/BLI-3, which generates ROS and is important for defense against bacterial pathogens	NOX1-5 and DUOX1-2 enzymes generate ROS; can have direct role in microbial killing in phagolysosome	Present with no known role in immunity	McCallum and Garsin, 2016; Hajjar et al., 2017
IFN-I response	No clear orthologs of ligands or receptors, but some triggers of IFN-I expression (e.g. RIG-I-like receptors) also trigger the IPR	Activated by RIG-I-like receptors; cGAS/STING	No clear orthologs	Lažetić and Troemel, 2020

bZIP, basic leucine zipper; DUOX, dual oxidase; IFN-I, type-I interferon; IPR, intracellular pathogen response; NLR, nucleotide-binding leucine-rich repeat; NOX, NADPH oxidase; ROS, reactive oxygen species; TIR-1, Toll/interleukin-1 receptor domain protein.

Innate immunity in many hosts can be triggered by detection of pathogen-associated molecular patterns (PAMPs), which are sensed by pattern-recognition receptors (PRRs), thereby activating downstream signaling. A classic example is detection of the Gram-negative bacterial PAMP lipopolysaccharide (LPS) by the mammalian PRR Toll-like receptor (TLR) 4, which activates NFκB to induce expression of pro-inflammatory cytokines. In contrast, *C. elegans* has only one TLR, the role of which in direct pathogen recognition remains unclear, and *C. elegans* has lost NFκB through evolution (Table 3) (Irazoqui et al., 2010b). So far, arguably the only known classic PAMP that is detected by both mammals and *C. elegans* is double-stranded RNA (dsRNA), a viral replication product PAMP, sensed by PRR RIG-I-like receptors (Table 3) (Lažetić et al., 2023a; Sowa et al., 2020; Ashe et al., 2013). A recent report found that C-type lectin receptor pairs, which in mammals detect pathogen-associated carbohydrates, are responsible in *C. elegans* for sensing oomycete species; however, the exact ligands sensed remain unknown (Table 3) (Liu et al., 2024).

Studies in *C. elegans* have uncovered new PAMPs and their recognition mechanisms in the host, such as a *P. aeruginosa*-associated virulence factor phenazine-1-carboxamide (PCN), which is sensed through the nuclear hormone receptor NHR-86/HNF4 (Table 3) (Peterson et al., 2023). Another study identified six volatile compounds produced by *P. aeruginosa* that are sensed by *C. elegans* neurons. These compounds included 1-undecene, which can induce immune genes and promote survival upon subsequent infection (Prakash et al., 2021), as well as systemic activation of the ER-UPR and increased lifespan (De-Souza et al., 2023). There are also many examples of immune responses in *C. elegans* being triggered by ‘patterns of pathogenesis’, which are common strategies and events used by pathogens to cause disease (Vance et al., 2009). These patterns include perturbations of proteostasis and other signs of infection that relate to ‘effector-triggered immunity’ (an innate immune response triggered by an effector protein from a pathogen), long appreciated in the plant immunity world and, more recently, in animal immunity (Tse-Kang et al., 2025; Remick et al., 2023).

Although there is evidence for pathogen-specific transcriptional responses, the limited mechanistic understanding of their activation and anti-microbial effects makes the distinction between stress response pathways and immune pathways in *C. elegans* less clear than in mammals. Thus, several proteostasis pathways in Table 1 could be listed also as immunity pathways in Table 3 and vice versa. Recent findings in the field show that proteostasis is not only targeted during infection but also serves as a crucial component of host defense. Therefore, in this Review we discuss proteostasis processes, including protein synthesis, folding, degradation and autophagy, how they are shaped by infection, and how proteostasis in turn influences immune responses. These processes are organized by the cellular compartment, beginning with the cytosol, followed by the endoplasmic reticulum (ER) and concluding with the mitochondria.

## Intersection between proteostasis pathways and immunity

### Pathways in the cytosol: protein synthesis

**Fig. 3.**
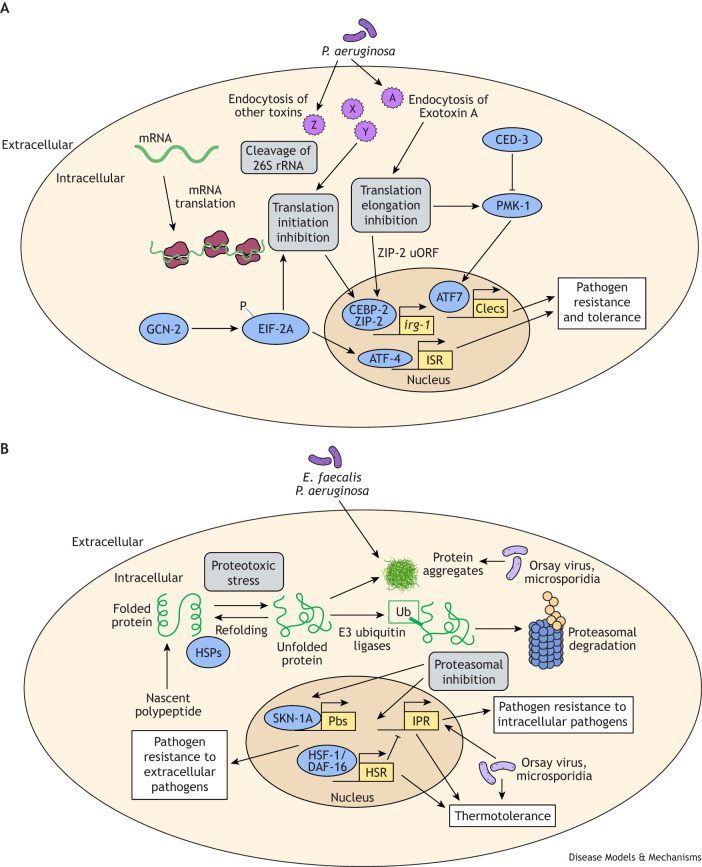
**Intersections between immunity and protein synthesis, heat shock resistance and folding proteasomal degradation.** (A) Blockade in translation initiation and elongation triggers immune responses. The EIF-2A kinase GCN-2 phosphorylates EIF-2A, which inhibits translation initiation, and activates ATF-4 and the integrated stress response (ISR). *P. aeruginosa* secretes many toxins including Exotoxin A, which is endocytosed into host cells and blocks translation elongation. This blockade is sensed by ZIP-2, as controlled by an upstream open reading frame (uORF). ZIP-2 acts with CEBP-2 to control *P. aeruginosa* response genes including *irg-1*. This blockade also triggers p38 MAPK PMK-1 signaling to transcription factor ATF7 to induce expression of genes including those encoding secreted C-type lectins (Clecs). *P. aeruginosa* infection also causes cleavage of 26S ribosomal RNA (rRNA) to block translation. The CEBP-2/ZIP-2 and ATF7 transcription factors promote pathogen resistance and tolerance. P, phosphorylation. (B) Roles of protein folding, temperature and proteasome in immune responses. The heat shock response (HSR) includes induction of heat shock proteins (HSPs) that act as chaperones to fold or refold proteins. Infection with extracellular pathogens such as *P. aeruginosa* and *E. faecalis*, as well as infection with intracellular pathogens can increase levels of protein aggregates. Infection with intracellular pathogens induce the intracellular pathogen response (IPR), which includes E3 ubiquitin ligase components that ubiquitylate proteins, often leading to degradation. Activation of the HSR or the IPR can lead to increased thermotolerance. Proteasomal inhibition activates expression of IPR genes, as well as SKN-1A-controlled genes, which include Pbs proteasomal subunits, which help restore proteasomal function. *E. faecalis* and *P. aeruginosa* are extracellular pathogens; Orsay virus and microsporidia are intracellular pathogens. Ub, ubiquitin. In A and B, shades of purple represent pathogens or pathogen-derived toxins (X, Y, Z, A), gray boxes represent perturbations in core processes, blue represents proteostasis machinery, yellow boxes represent transcriptional defense pathways, and white boxes represent outcomes.

Proteostasis begins in the cytosol with protein synthesis, coordinated by the translational machinery, which includes initiation and elongation factors that aid ribosomes in translating mRNA into protein (Fig. 3A). In this section, we cover how blockade of either translation initiation or elongation impact immune responses and survival upon infection in *C. elegans*.

A major anti-viral strategy in mammals is the integrated stress response (ISR), which blocks translation initiation to prevent viruses from using host translation machinery to synthesize their own proteins (Ito et al., 2023). Translation can be halted by specialized kinases that sense stress and then trigger the ISR by phosphorylating and inactivating eukaryotic initiation factor 2a (EIF2A) (Table 1). During viral infection, protein kinase R (PKR) senses viral dsRNA to activate the ISR upon viral infection. Notably, *C. elegans* lacks PKR, although it does have the EIF-2A kinase called general amino acid nonderepressible 2 (GCN-2), which senses nutrient deprivation (Hinnebusch, 2005). Recent findings in *C. elegans* demonstrated that infection with *Salmonella enterica* induces the GCN-2/EIF2A signaling pathway to activate the downstream transcription factor ATF-4 (Wibisono et al., 2025). However, ATF-4 impairs survival upon infection, perhaps through its suppression of ribosomal protein expression. In a similar vein, intestinal defense against *P. aeruginosa* has been shown to benefit from increased expression of protein synthesis factors such as ribosomal protein RPS-1 and translation initiation factor EIF-3.J (Liu et al., 2016). In both of these cases, the host-directed blockade in translation initiation appears to be detrimental during bacterial infection.

Several studies have investigated pathogen-mediated blockade of host translation elongation, in which the *C. elegans* host response appears to be beneficial. Given that immune responses rely on synthesizing proteins such as anti-microbial peptides, this process is highly targeted by pathogenic microbes, including *P. aeruginosa*. For example, *P. aeruginosa* produces Exotoxin A, which is endocytosed into host cells of mammals and *C. elegans* to inactivate eukaryotic elongation factor 2 (EF2) and block host translation elongation (Fig. 3A) (Haag et al., 2014; McEwan et al., 2012; Dunbar et al., 2012; Troemel, 2012). Genetic studies of the interaction between Exotoxin A and EF2 in *C. elegans* indicate that the host response is triggered not by the direct recognition of the toxin shape but rather by the recognition of its activity in blocking translation elongation (McEwan et al., 2012), consistent with a ‘pattern of pathogenesis’.

*C. elegans* responds to Exotoxin A through multiple transcriptional pathways. These pathways include the PMK-1/p38 MAPK pathway (McEwan et al., 2012), which plays a central role in controlling *C. elegans* defense against many pathogens (Table 3, Fig. 3A). Acting in parallel to the PMK-1 pathway, two basic leucine zipper (bZIP) transcription factors, CEBP-2 and ZIP-2, control upregulation of defense genes (Estes et al., 2010; Reddy et al., 2016). CEBP-2 and ZIP-2 likely form a heterodimer (Reinke et al., 2013) to control induction of *irg-1*, a common reporter for the *C. elegans* transcriptional response to *P. aeruginosa*, as well as several other *P. aeruginosa* response genes in response to Exotoxin A (Table 3, Fig. 3). ZIP-2 and CEBP-2 are both required for controlling pathogen load and survival upon *P. aeruginosa* infection; furthermore, they both promote survival when animals are exposed to Exotoxin A in the absence of infection (Dunbar et al., 2012; Reddy et al., 2016; McEwan et al., 2012). Although CEBP-2 protein is constitutively present (Reddy et al., 2016), ZIP-2 protein synthesis appears to be repressed via an upstream open reading frame (uORF) that overlaps with the predicted start codon for ZIP-2, and this repression is relieved to induce expression upon infection (Dunbar et al., 2012). Intriguingly, recent findings indicate that the uORF may become fused with the canonical open reading frame through ribosomal frameshifting (Kniazeva and Ruvkun, 2025). Although the exact mechanistic details are unclear, altogether these results indicate that ZIP-2 is both a sensor and a signaling molecule for immune response to translation elongation block during infection.

Many studies demonstrate that virulence factors are redundant, as removing a single factor does not impair pathogen virulence in a host (Ghosh and O'Connor, 2017). Although *P. aeruginosa* Exotoxin A mutants no longer cause translational block in the *C. elegans* intestine, these mutants still induce *irg-1* and still kill worms (i.e. Exotoxin A is sufficient to induce *irg-1* and kill hosts, but not necessary) (Dunbar et al., 2012; McEwan et al., 2012). The remaining pathogenicity of Exotoxin A mutants, and their ability to induce *irg-1*, is likely due to the existence of other virulence factors, potentially those that block other core host processes (Dunbar et al., 2012; Melo and Ruvkun, 2012). In addition to blocking translation elongation, it was discovered in *C. elegans* that infection with *P. aeruginosa* also causes cleavage of 26S ribosomal RNA. This effect is dependent on endocytosis, but independent of Exotoxin A, indicating that it is controlled by another virulence factor (Fig. 3A) (Vasquez-Rifo et al., 2020). That said, this effect is dependent on the upstream response regulators GacA/GacS, which control an armamentarium of virulence factors and much of *P. aeruginosa* pathogenesis in many hosts including *C. elegans*.

In addition to studying the pathogen-induced impacts on host translation and resulting defense responses, a recent study deliberately perturbed translation and then studied the impact on defense responses. Here, the authors found that blockade in translation initiation was protective in controlling pathogen load and improving survival upon *P. aeruginosa* infection, while blockade in translation elongation was protective in controlling pathogen load but detrimental to overall survival (Ghosh and Singh, 2024). This study reported that ZIP-2 controls a response to translation initiation, in contrast to other studies that indicated that ZIP-2 is not required for response to blockade in translation initiation (Dunbar et al., 2012; Kniazeva and Ruvkun, 2025). Further work will be required to understand how blockade of initiation is beneficial in this context (Ghosh and Singh, 2024), in contrast to the studies mentioned above, in which it appears to be detrimental (Wibisono et al., 2025; Liu et al., 2016). Nonetheless, the findings illustrate how blockade in mRNA translation can provide an immune benefit to hosts.

An interesting and unanswered question is, how do host cells overcome pathogen-induced blockade of translation to produce immunity proteins? Similarly to studies in *C. elegans*, studies in mammalian cells have demonstrated that pathogen-derived translation-blocking toxins induce innate immune responses, including the induction of cytokines (Fontana et al., 2011). Notably, in order for mouse macrophages to produce cytokine proteins in the context of translational blockade by *Legionella pneumophila* (a bacterium that causes Legionnaire's disease, a type of pneumonia), superinduction of mRNAs was required (Barry et al., 2017). Indeed, in *C. elegans* studies, transcriptional responses to *P. aeruginosa* infection involve genes such as *irg-1* induced at ∼1000× over uninfected levels (Estes et al., 2010). Other studies of *L. pneumophila* infection that discriminated between the responses of infected and bystander cells provided insight into how defense proteins can be produced in the context of translation-blocking toxins (Copenhaver et al., 2015). Specifically, it was discovered that infected mouse macrophages could only produce IL-1 ligands, which interacted with IL-1 receptors on neighboring cells. In turn, bystander cells robustly produced cytokines such as TNF-alpha (also known as TNF), IL-6 and IL-12 required for protection, which the infected cells themselves could not produce. Future studies distinguishing responses in infected and uninfected cells in *C. elegans* can help determine how immune proteins are produced despite the translation-blocking effects of pathogens such as *P. aeruginosa*.

In summary, studies in *C. elegans* have provided insights into how blockade in translation elongation and translation initiation relate to immunity. Regarding translation initiation, there are mixed results showing that blockade can be beneficial or detrimental during bacterial infection, which will need to be clarified. It would be interesting to explore in the future how blockade in translation initiation impacts viral infection in *C. elegans*, given its benefit in mammalian infections via ISR activation through the PKR sensor, which is lacking in *C. elegans*. Although the ISR is thought to restrict viral infection and alleviate proteostatic burden during stress, dysregulated ISR may contribute to diseases including cognitive disorders, neurodegeneration, cancer, diabetes and metabolic disorders (Costa-Mattioli and Walter, 2020). There are a variety of ISR activators and inhibitors that are being tested in the clinic for treating human disease including neurodegenerative diseases. Thus, careful regulation of this response, as well as other translational responses, are crucial to promote host survival upon infection without causing collateral damage. Regarding elongation studies in *C. elegans*, ZIP-2 is a defense transcription factor that uses its overlapping uORF as a sensor to detect a blockade in translation elongation. Humans do not have a direct ortholog of ZIP-2. However, there is a direct ortholog of the ZIP-2 heterodimeric binding partner CEBP-2, called CEBP-gamma in mammals. CEBP-gamma has many bZIP transcription factor binding partners (Reinke et al., 2013), including those with overlapping uORFs such as CEBP-beta (Wethmar et al., 2010), which has a role in inflammation (Ren et al., 2023). Future studies could investigate whether such bZIP transcription factors in mammals use uORFs as sensors for pathogen-induced blockade in translation elongation.

### Pathways in the cytosol: protein folding and heat shock response

Having considered interconnections between *C. elegans* immunity and protein synthesis, we next consider connections between *C. elegans* immunity and protein folding. After proteins are synthesized, they need to fold into their proper three-dimensional structure, which is facilitated by specialized proteins called molecular chaperones (Fig. 3B). In addition to their role in facilitating native protein folding, chaperones also aid in the refolding of aggregated or misfolded proteins caused by stressors, such as heat shock (Singh et al., 2024). When proteins become irreparably misfolded, molecular chaperones can direct them to cellular degradation pathways such as autophagy and the ubiquitin–proteasome system. Cytosolic chaperones include HSP90 and the HSP70 family, as well as small heat shock proteins (sHSP) such as the HSP16 family, which are induced after heat shock conditions (Brehme et al., 2014). Induction of these heat shock proteins (HSPs) is mediated by heat shock factor (HSF-1), a transcription factor that acts as a master regulator of the cytosolic heat shock response (HSR) and is conserved from yeast to humans (Table 1) (Anckar and Sistonen, 2011; Li et al., 2017).

*C. elegans* provides a particularly useful model for understanding the role of HSF-1 and the HSR in aging and immunity in metazoans, given that HSF-1 and HSPs are conserved (Table 1). In *C. elegans* it has been shown that HSF-1 contributes to longevity and to the regulation of immune responses to infection. In pioneering studies on *C. elegans* lifespan mediated by insulin-like growth factor receptor DAF-2 and its downstream transcription factor DAF-16/FOXO, it was found that DAF-16 functions together with HSF-1 to increase lifespan (Kenyon et al., 1993; Hsu et al., 2003; Morley and Morimoto, 2004; Chiang et al., 2012). Subsequent studies at the intersection of proteostasis and immunity in *C. elegans* showed that the DAF-2/DAF-16 signaling pathway regulates immunity against *P. aeruginosa*, *E. faecalis* and *S. enterica* through HSF-1 and a system of HSP chaperones (Garsin et al., 2003; Singh and Aballay, 2006). In turn, heat shock was shown to increase survival upon *P. aeruginosa* infection in an *hsf-1*-dependent manner, and independently of the PMK-1/p38 MAPK pathway. Similar effects were observed with HSF-1 overexpression in the absence of heat shock (Singh and Aballay, 2006) (Fig. 3B). Consistent with transcriptional profiling, lifespan and pathogen survival studies, these findings indicate that the DAF-2/DAF-16/HSF-1 pathway acts in parallel to PMK-1/p38 MAPK signaling (Troemel et al., 2006).

Pathogen infection can cause proteotoxic stress in *C. elegans* and humans, which can manifest as protein misfolding and aggregation (Bakowski et al., 2014; Aviner and Frydman, 2020). This misfolding is sometimes related to production of reactive oxygen species (ROS), either through dysfunction in the mitochondrial electron transport chain, or by ROS specifically generated by specialized enzymes as part of the host response (Table 3) (Goswamy and Irazoqui, 2021). Aggregated proteins are a hallmark of neurodegenerative diseases such as Huntington disease, which is caused by expanded glutamine (polyQ) repeats in the huntingtin protein. An increased number of glutamine repeats, with Q35 as a threshold number, enhances the likelihood of early protein aggregation and disease onset (Morley et al., 2002). Many interesting studies in *C. elegans* have investigated the impacts of the microbiome or probiotics on protein aggregates and heterologous polyQ expression in neurons, muscle and the intestine itself (Walker et al., 2022, 2024; Goya et al., 2020). In this Review, we focus only on protein aggregate studies involving pathogens and measurements of immune responses or immune pathways.

*C. elegans* infections caused by several bacterial pathogens, including *P. aeruginosa* and *E. faecalis*, result in an increase in polyglutamine aggregates in the intestine. This process can be controlled by the DAF-2,16/HSF-1 axis (Fig. 3B). This protective effect is mediated by multiple HSPs and oxidative stress enzymes (Mohri-Shiomi and Garsin, 2008). More recently, it has been shown that the decreased survival of older animals upon infection with *P. aeruginosa* (immune aging) is also regulated by DAF-2,16/HSF-1. In particular, DAF-16/HSF-1 and ZIP-10 transcription factors regulate INS-7, an insulin-like growth factor for which age-dependent increase leads to immune aging (Lee et al., 2021). Thus, it appears that insulin-like growth factor signaling in wild-type animals causes immune aging through a feedforward mechanism involving the proteostasis factor HSF-1. Another study found that loss of HSF-1 in younger, but not older, *C. eleg*ans triggered several compensatory responses, including ER-UPR, the SKN-1-mediated oxidative response and the intracellular pathogen response (IPR), leading to increased tolerance of heat shock (Kovács et al., 2024), which is surprising as loss of HSF-1 is normally expected to decrease tolerance of heat shock.

Having discussed the intersection of pathogen infection, proteostasis, immune and stress responses in *C. elegans* during extracellular infection, we now turn to intracellular infection. The Orsay virus and microsporidia are the only pathogens known to replicate intracellularly in the *C. elegans* intestine (Balla and Troemel, 2013). Infection with these two pathogens causes the accumulation of large aggregates of the protein ubiquitin later during infection (Bakowski et al., 2014), indicating that, like extracellular infections, intracellular infections also cause proteotoxic stress (see the ‘Pathways in the cytosol: ubiquitin–proteasome system and other protein degradation’ section for more information on ubiquitin). In response to these intracellular infections, *C. elegans* upregulates a transcriptional response that is distinct from the response to extracellular pathogens (Bakowski et al., 2014). The Orsay virus and *N. parisii* are different types of pathogens (Table 2), but they trigger transcriptional upregulation of a similar set of genes in *C. elegans* known as the IPR genes. Activation of the IPR provides this organism with resistance against intracellular infection as well as increased tolerance of 37°C heat shock (thermotolerance) (Fig. 3B) (Reddy et al., 2017, 2019). For context, *C. elegans* normally grows at temperatures between 16°C and 25°C. The IPR shares many genes in common with those induced by chronic heat stress at 30°C but not the sHSPs, which are induced at higher temperatures by HSF-1 (Bakowski et al., 2014; Reddy et al., 2017). This observation suggests that the IPR is a protective response separate from the HSR, with the IPR triggered by longer exposure at temperatures around 30°C (in addition to being triggered by intracellular infection), compared to the HSR triggered by shorter exposure at temperatures such as 33-37°C (Zevian and Yanowitz, 2014; Golden et al., 2020; Bar-Ziv et al., 2020b). Further research has shown that HSR and IPR are two distinct pathways. In particular, loss of the IPR negative regulator PALS-22 combined with loss of HSF-1 causes larval arrest (Reddy et al., 2017), perhaps because IPR genes are independently upregulated in *hsf-1* mutants (Kovács et al., 2024). High upregulation of IPR genes has been shown to cause larval arrest (Lažetić et al., 2023b). These observations suggest that there is an antagonistic relationship between the HSR and IPR (Fig. 3B).

Several studies have indicated further connections between viral infection and heat shock. For example, it has been shown that exposure to 35°C heat shock increases protection against viral infection (Huang et al., 2021), similarly to protection against bacterial infection. Decreased viral load is seen if the heat shock is applied before or 3 h after viral inoculation, alleviating concerns that it could simply be due to heat shock impairing feeding and, thus, animals receiving a lower viral inoculation. Interestingly, viral infection provides protection against heat shock, which correlated with mRNA expression of the argonaut protein genes *alg-1* and *alg-2* (Castiglioni and Elena, 2024). Also, in the absence of infection, activation of the cytosolic sensor for the Orsay virus, RIG-I/DRH-1, can induce IPR gene expression as well as increased thermotolerance (Batachari et al., 2024a). The mechanisms at play for these interactions between the Orsay virus, its sensor and heat are areas for future research.

In summary, work in *C. elegans* has shown that heat shock promotes resistance to both extracellular bacterial infection and intracellular viral infection, and both of these infection types can cause proteotoxic stress manifested by build-up of protein aggregates. Furthermore, HSF-1, HSPs and the HSR appear to mediate the increased resistance to bacterial infection, including control of protein aggregates formed by infection. Notably, insulin signaling in older *C. elegans* impairs bacterial immunity in a manner involving HSF-1, which would be interesting to explore further in mammals, which have homologs of all these factors. In mammals, HSF1 and the HSR appear to have mixed effects on immunity. For example, in some viral infections, HSF1 appears to benefit the host, whereas in other viral infections HSF1 benefits the virus (Reyes et al., 2022; Pauciullo et al., 2024). Several studies indicate that HSF1 opposes TNF-alpha-induced NFκB innate immune signaling, which promotes anti-bacterial and anti-viral immunity (Paszek et al., 2020; Shi et al., 2022). This inhibitory effect of HSF1 on TNF-alpha/NFκB immune signaling in mammals has conceptual similarities to HSF-1 repressing the IPR in *C. elegans*. Given that *C. elegans* lacks TNF-alpha/NFκB signaling, the mechanisms underlying these interactions that lead to growth arrest are likely to be novel and could be further explored in mammals as details are uncovered. The mechanisms of the IPR are discussed in the sections below on the proteasome and lysosomes, which control protein degradation.

### Pathways in the cytosol: ubiquitin–proteasome system and other protein degradation

Proteins are targeted for degradation if they cannot be refolded and are irreparably damaged; inhibitor proteins can also be targeted for degradation in order to activate downstream signaling. Protein degradation and its intersections with immunity will be considered in this section. Arguably, the major protein degradation pathway inside cells is the ubiquitin–proteasome system. This system includes E3 ubiquitin ligases that catalyze the covalent attachment of a ubiquitin tag to substrate proteins, a process called ubiquitination or ubiquitylation (we use the latter term in this Review) to direct their degradation by the proteasome (Fig. 3B). Ubiquitylated proteins can also have non-proteasomal fates, including degradation of larger substrates by the autophagy/lysosome system (see ‘Pathways in the cytosol: autophagy into lysosomes’ section). However, degradation by the proteasome is thought to be the major destination for ubiquitylated substrates, especially smaller ones. The proteasome plays a key role in several immune responses, e.g. it is required to activate NFκB signaling in mammals owing to proteasomal degradation of the IκB inhibitor. When pathogens block proteasome function, hosts can detect this disruption and rapidly upregulate defense pathways (for a review of information specifically on ubiquitin-related processes and *C. elegans* immunity, see Garcia-Sanchez et al., 2021). Here, we focus on where ubiquitin and the proteasome intersect with innate immune signaling and proteostasis.

A transcriptional analysis of the early *C. elegans* response to proteasomal blockade by the chemical inhibitor bortezomib revealed induction of two non-overlapping gene sets (Fig. 3B) (Reddy et al., 2019). The first gene set includes genes induced by the transcription factor SKN-1, including proteasomal subunits as part of a cellular feedback response discussed below in the context of pathways associated with the ER. The second gene set includes genes induced as part of the IPR mentioned above. Induction of the IPR is conserved across different *Caenorhabditis* host species and *Nematocida* pathogen species (Bakowski et al., 2014; Chen et al., 2017b; Wan et al., 2022). Despite being induced by proteasome blockade, IPR genes do not include proteasome subunits, but rather include cullin-ring ubiquitin ligase components, which have expanded evolutionarily in the lineage that gave rise to *C. elegans*, with 300-500 F-box proteins that serve as adaptors to identify substrates in comparison to 69 in humans (Bosu and Kipreos, 2008; Thomas, 2006). *C. elegans* also has an expanded family of F-box-binding proteins called Skp-related protein genes (SKRs), with 23 genes found in *C. elegans* compared to one in humans. SKRs bind to cullins, which bind to RING-domain proteins that bring in an E2 ubiquitin ligase charged with a ubiquitin that is transferred to a substrate identified by the F-box protein. Initial descriptions of the expanded cullin-ring ubiquitin ligase family in *C. elegans* proposed that the large number of ubiquitin ligase adaptors could be used to target intracellular pathogens and their proteins for destruction (Thomas, 2006), although evidence for this function is still scarce, perhaps due to functional redundancy, which makes it difficult to analyze with loss-of-function genetics (Lažetić and Troemel, 2020).

There is strong evidence for a cullin-ring ubiquitin ligase in *C. elegans* promoting increased proteostasis, as measured by increased thermotolerance, as well as a decrease in protein aggregates (Reddy et al., 2017). Through a combination of genetics and biochemistry, a previously uncharacterized cullin-ring ubiquitin ligase complex composed of two F-box proteins, three SKRs, a RING-domain protein and CUL-6/cullin was shown to be activated as part of the IPR (Reddy et al., 2017; Panek et al., 2020). CUL-6/SKR-3,4,5 have a minor role in pathogen resistance against *N. parisii* and virus infection (Bakowski et al., 2014; Panek et al., 2020; Reddy et al., 2017), but are required to promote thermotolerance when the IPR is activated. The fate of substrates targeted by this CUL-6 ubiquitin ligase appears to be the lysosome, as discussed in the ‘Pathways in the cytosol: autophagy into lysosomes’ section, even though its expression, as well as that of other IPR genes, is strongly upregulated by blockade of the proteasome.

The IPR is induced upon viral infection via DRH-1 sensing of viral RNA, and a recent study demonstrates that DRH-1 can be degraded by the proteasome, which impairs IPR induction (Zhang and Samuelson, 2025). This proteasomal degradation of DRH-1 increases as animals age, likely owing to dysregulated SUMOylation, which is a ubiquitin-like modification that can precede proteasomal degradation. A surprising and separate role was found for the 19S proteasomal subunit RPT-6 in defense against *P. aeruginosa* infection. Specifically, RPT-6 was found as a binding partner for the GATA transcription factor ELT-2, which controls immune gene expression in the gut and survival upon *P. aeruginosa* infection (Olaitan and Aballay, 2018). Here, RPT-6 functioned together with ELT-2 to promote gene expression and increased survival. Interestingly, this role for RPT-6 appeared to be independent of its canonical proteasomal role in protein degradation, providing a distinct example of how proteasome machinery and immunity can be interlinked.

One protein degradation event that occurs independently of the proteasome and is related to immunity is the inactivating cleavage of PMK-1/p38 MAPK by the CED-3 caspase required for apoptosis, programmed cell death, although in this case CED-3 appears to act independently of apoptosis (Fig. 3A) (Weaver et al., 2020). Thus, here, the overall impact of CED-3 is to inhibit immunity, in contrast to mammalian immunity, in which caspases limit viral infection by apoptosis of infected cells or process pro-inflammatory cytokines such as IL-1beta, and in both cases promote immunity. In *C. elegans*, CED-3 has so far not been shown to limit infection through apoptosis of infected cells. That said, CED-3 has been reported to promote increased resistance to vaccinia virus infection through unknown mechanisms (Liu et al., 2006), and also promotes increased survival upon *S. enterica* infection (Aballay and Ausubel, 2001; Aballay et al., 2003). It will be interesting to explore how these findings connect with the CED-3-mediated cleavage of PMK-1, as other studies have highlighted how PMK-1 controls expression of proteostasis genes, including those involved in ubiquitylation-related processes and the lysosome (Yuan et al., 2023; Balasubramaniam et al., 2022).

In summary, there are several connections between *C. elegans* immunity, ubiquitin ligases, protein degradation and proteostasis. First, there is a robust transcriptional response to blockade of the proteasome, including SKN-1A-controlled proteasome subunits, and IPR genes, such as those that encode components of a CUL-6 ubiquitin ligase. This CUL-6 ubiquitin ligase serves to restrict protein aggregates and promote thermotolerance, with a minor role in immunity. Interestingly, there are several human variants in proteasome subunits associated with hyperinflammatory diseases called interferonapathies that are distinguished by high levels of type-I interferons (IFN-Is), which activate the major innate immune pathway against intracellular pathogens (Lažetić et al., 2023a). Of note, activation of the IFN-I response in mammals has many similarities with activation of the IPR in *C. elegans*, so there may be connections between how blockade in the proteasome regulates intracellular immune responses in mammals and *C. elegans*. It would be interesting to determine whether RIG-I has increased degradation by the proteasome upon aging in mammals, similar to DRH-1 degradation in older *C. elegans*. Other protein degradation/immune findings from *C. elegans* have demonstrated a non-canonical role for CED-3/caspase in suppressing immune signaling, and a non-canonical role for a proteasomal subunit in promoting immune signaling, both of which could be explored in mammals owing to the presence of orthologs of CED-3.

### Pathways in the cytosol: autophagy into lysosomes

As mentioned above, ubiquitylation of smaller substrates such as individual proteins or peptides will lead to their degradation by the proteasome, whereas larger substrates are degraded through autophagy (self-eating), which is covered in this section. These larger substrates can include multi-subunit protein machines, such as the ribosome; entire organelles, such as mitochondria; and intracellular pathogens (Dikic, 2017). Many pathogens will block and/or hijack autophagy to promote their survival and replication (Kuo et al., 2018). Although a wide range of autophagy-related processes has now been described, this Review focuses on canonical macroautophagy (hereafter referred to as autophagy). This process involves engulfing material from the cytosol within a newly generated membrane-bound compartment called the autophagosome, which then fuses with the lysosome for degradation of the contents. When autophagy is used to degrade intracellular pathogens, it is called ‘xenophagy’ (foreign eating).

*C. elegans* has many of the same canonical autophagy components found in other eukaryotes such as yeast. Several metazoan-specific components have been identified in this organism through genetic screens (Table 1) (Zhang and Melendez, 2025; Kuo et al., 2018). However, very little is known about xenophagy in *C. elegans*, although it is attractive to speculate that it would be a key defense strategy given that *C. elegans* has non-renewable cells and does not appear to clear infected cells through apoptosis in response to infection, as mentioned above. Early studies suggested that *C. elegans* intestinal cells used autophagy to clear intracellular *S. enterica*, a facultative intracellular pathogen in humans (Jia et al., 2009). However, subsequent studies have failed to confirm that *S. enterica* resides intracellularly within intestinal cells and thus have been unable to reproduce the autophagy-dependent clearance observed in earlier reports (Curt et al., 2014).

**Fig. 4.**
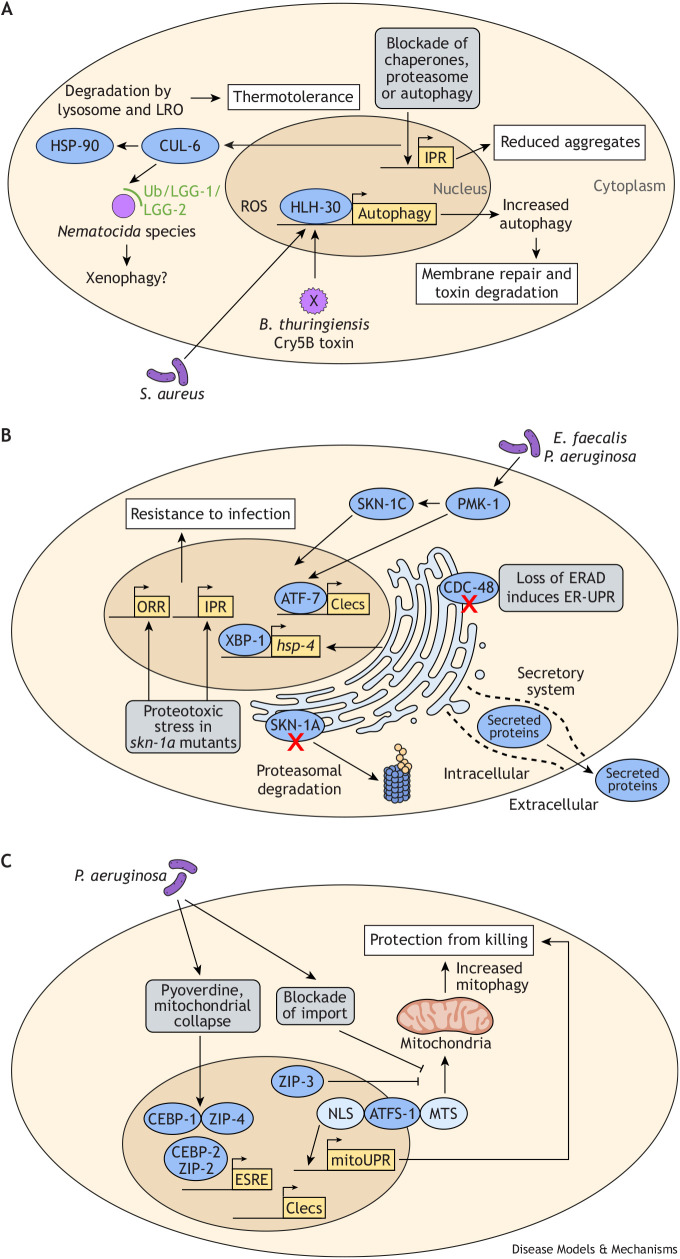
**Intersections between immunity and membrane-associated proteostasis pathways, including lysosomes, endoplasmic reticulum and mitochondria.** (A) CUL-6 ubiquitin ligase targets HSP-90 for degradation by lysosomes and lysosome-related organelles (LROs), promoting thermotolerance. CUL-6 also targets intracellular *Nematocida* microsporidia species along with autophagy components LGG-1/LGG-2 for xenophagy. The redox-sensitive transcription factor HLH-30 induces autophagy in response to extracellular pathogens such as *S. aureus*, which can boost immunity as shown against *B. thuringiensis* Cry5B toxin damage. Blockade of chaperones, proteasome or autophagy activates the intracellular pathogen response (IPR) in the intestine and can lead to reduced protein aggregates through a compensatory cross-tissue protective response. (B) The endoplasmic reticulum (ER)-associated isoform SKN-1A is tethered to the ER and constitutively degraded by the proteasome via ER-associated degradation (ERAD) under basal conditions. Upon proteasomal blockade, SKN-1A transits to the nucleus and activates proteasome subunit expression (Fig. 3B). Loss of SKN-1A causes proteotoxic stress that constitutively activates the IPR and oomycete recognition response (ORR), promoting resistance to microsporidia and oomycete pathogens. Loss of SKN-1C causes susceptibility to infection by bacterial pathogens *E. faecalis* and *P. aeruginosa* downstream of reactive oxygen species (ROS) generation. ROS activates the PMK-1/p38 MAPK pathway, a key player in triggering defense gene expression [e.g. Clec genes (Clecs)]. The endoplasmic reticulum unfolded protein response (ER-UPR; shown as *hsp-4* reporter induction), activated by XBP-1, is essential for larval development during *P. aeruginosa* infection. Loss of XBP-1 can be compensated by ERAD activation. Conversely, loss of CDC-48, an ERAD component, induces ER-UPR through IRE-1, which blocks the SKN-1C response. (C) Under normal conditions, ATFS-1 enters mitochondria via its mitochondrial targeting sequence (MTS), where it is degraded. Upon mitochondrial stress or pathogen infection with *P. aeruginosa*, mitochondrial import is blocked, and ATFS-1 translocates to the nucleus via its nuclear localization sequence (NLS), inducing expression of mitochondrial chaperones (*hsp-6*, *hsp-60*), as part of the unfolded protein response in mitochondria (mitoUPR), as well as innate immune genes including Clecs. *P. aeruginosa*-derived toxins such as pyoverdine cause mitochondrial collapse, activating the ethanol and stress response element (ESRE) network controlled by basic leucine zipper (bZIP) transcription factors CEBP-1, CEBP-2, ZIP-2 and ZIP-4. ZIP-3 inhibits ATFS-1 during phenazine-associated *P. aeruginosa* infections. Mitophagy (autophagy of damaged mitochondria) provides protection against pathogen-induced mitochondrial damage and killing. In A-C, purple represents pathogens or a pathogen-derived toxin (X), gray boxes represent perturbations in core processes, blue represents proteostasis/immunity machinery, yellow boxes represent transcriptional defense pathways, and white boxes represent outcomes. Ub, ubiquitin.

So far, there are no bacteria convincingly shown to replicate inside intact *C. elegans* cells. However, there are many species from the Microsporidia phylum of intracellular fungi shown to replicate inside intact *C. elegans* cells and spread throughout various organs, with *N. parisii* being the best-studied example (Gang and Lažetić, 2024; Tecle and Troemel, 2022). Here, ubiquitin and autophagy factors were found in proximity to *N. parisii* sporoplasms (Fig. 4A). These factors include the early marker LGG-1/Atg8, which is commonly a precursor to engulfment by autophagosomes and degradation by lysosomes, as well as CUL-6, mentioned earlier (Bakowski et al., 2014). Indeed, this study also demonstrated that autophagy functionally served to reduce *N. parisii* load. In studies of natural host/pathogen variation, it was found that another microsporidia species, *Nematocida ironsii*, is better targeted by LGG-2/Atg8 in *C. elegans* wild isolate CB4856 from Hawaii than in the N2 wild-type strain from England. CB4856 animals can clear *N. ironsii* infection, unlike N2 animals (Balla et al., 2015). Further analysis is needed to better understand whether canonical xenophagy is involved here (Balla et al., 2019).

The studies mentioned above indicated that a CUL-6 ubiquitin ligase may target *N. parisii* for degradation, but this ubiquitin ligase also has a role in the absence of infection, whereby it promotes increased thermotolerance. Recently, it was found that one likely substrate for a CUL-6 ubiquitin ligase in this context is HSP-90 (Fig. 4A) (Bardan Sarmiento et al., 2024). Unlike many other HSPs that are expressed at low levels in the absence of heat shock, HSP-90 is a large constitutively expressed protein that is required for the activity of ∼20% of all proteins and 60% of all kinases (van Oosten-Hawle, 2023; Taipale et al., 2010, 2012). A CUL-6 ubiquitin ligase appears to target HSP-90 for destruction by lysosomes and lysosome-related organelles (LROs), specifically in the intestine (Fig. 4A) (Bardan Sarmiento et al., 2024). Prior studies in human cells indicated that inhibition or depletion of HSP90 could promote thermotolerance because it enabled HSF1 to induce the HSR (Zou et al., 1998). However, the CUL-6-mediated effect on thermotolerance in *C. elegans* appeared to be independent of HSF-1. Correspondingly, prior work had demonstrated that knockdown of HSP-90 in the intestine could induce HSP-70 in muscle cells, which was also independent of HSF-1 and relied on a homeodomain transcription factor to regulate HSP-70 expression and thermotolerance (Miles et al., 2023; van Oosten-Hawle et al., 2013). For future work, it would be interesting to investigate whether CUL-6 activation causes signaling to non-intestinal tissues.

As noted, CUL-6-mediated degradation of HSP-90 appeared to be directed to the lysosome or LROs, which are a prominent feature in the *C. elegans* intestine. Although lysosomes have a clear degradative function, it is less clear whether LROs play such a role. Both lysosomes and LROs can be targeted by pathogens, and their inhibition appears to trigger protection (Hajdú et al., 2023). Interestingly, one mechanism by which *C. elegans* may sense these impacts is through monitoring the deacidification of LROs. When under pathogen attack, LROs become more alkaline and shrink, which causes multimerization of the TIR-domain TIR-1 protein that activates ATF-7/p38 MAPK signaling in defense against *P. aeruginosa* infection (Tse-Kang et al., 2024). Autophagy may also serve to inhibit the damaging effects of necrosis during *P. aeruginosa* infection to improve defense against this pathogen (Zou et al., 2014).

Even when pathogens remain extracellular, the lysosome and autophagy can have important roles. For example, the bacterial pathogen *S. aureus* causes a lethal intestinal infection in *C. elegans* but appears to be entirely extracellular (Irazoqui et al., 2010a). Infection with *S. aureus* induces protective expression of many autophagy and lysosomal-related genes, controlled by the helix-loop-helix transcription factor HLH-30 (Fig. 4A). Interestingly, HLH-30 promotes immunity against *S. aureus* in *C. elegans.* Similarly, its homolog TFEB promotes immunity in mammalian cells (Lapierre et al., 2013; Visvikis et al., 2014; Najibi et al., 2016). HLH-30 appears to be regulated by a redox-dependent mechanism involving ROS that control its nuclear localization (Colino-Lage et al., 2024). For further information on the topic of how ROS generated in the context of infection could lead to a collapse in proteostasis, please see a review by Goswamy and Irazoqui (2021). A separate role for autophagy components has been shown for the *C. elegans* response to the Cry5B pore-forming toxin made by the extracellular bacterium *Bacillus thuringiensis* that compromises the integrity of the apical membrane of intestinal cells. Here, autophagy components appear to both target the Cry5B protein for degradation and repair the membrane, as part of an HLH-30-mediated response (Chen et al., 2017a) (Fig. 4A). Interestingly, HLH-30 promoted *C. elegans* tolerance against infection with enterohemorrhagic *E. coli* O157:H7 (EHEC), improving survival in the context of mutations that increased pathogen load (Tsai et al., 2021). These studies highlight an example of a host factor that acts to lower pathogen levels but ultimately has a detrimental effect on host physiology.

Paradoxically, many examples described in the sections above suggest that impairment of one host proteostasis or defense pathway can improve health, which appears to be due to compensatory responses. For example, a recent study demonstrated that impairment of any of the three canonical proteostasis pathways in *C. elegans* led to cross-tissue protective responses (Jung et al., 2023). Specifically, impairment of either autophagy, chaperones or the proteasome in the body wall muscle led to increased protein aggregates in that tissue but a reduction in aging-associated protein aggregates in the pharynx, which is the feeding organ of *C. elegans* (Fig. 2, Fig. 4A). This compensatory effect was independent of canonical autophagy components but did depend on the lysosome, perhaps due to a variant of autophagy called microautophagy, a process of the lysosome engulfing cargo with vesicles formed at its surface or by late endosomes (Wang et al., 2023). The safety mechanism appeared to involve IPR genes including the gene encoding PALS-5 protein, which localized to protein aggregates in the pharynx and played a role in preventing their aggregation (Jung et al., 2023).

In summary, the lysosome and autophagy machinery promote resistance to infection by intracellular pathogens such as microsporidia, as well as extracellular pathogens such as *S. aureus* and *E. coli*. The IPR, a transcriptional response to intracellular pathogens, promotes improved proteostasis, through targeting HSP-90 to the lysosome. The IPR and the lysosome may also be involved in a surprising benefit whereby aging-associated protein aggregates are prevented from forming in the *C. elegans* pharynx, if there is inhibition of autophagy, the proteasome or the HSR. These findings on new ways in which the lysosome may provide protection against protein aggregates could be of particular interest for understanding the mechanisms and treatment of aging-associated neurodegenerative diseases, such as AD in humans. Lysosomal dysfunction has been implicated in AD, with promising pharmacological treatments directed at improving lysosomal dysfunction such as those targeting TFEB (Kim et al., 2025).

### Pathways associated with the ER

Having considered intersections between immunity and homeostasis of proteins in the cytosol, and those degraded by the lysosome, we now turn to secreted proteins, which usually travel through the ER before being released into extracellular space. Secretion of immune factors is thought to pose a significant stress on the ER, which leads to misfolded proteins in this organelle, triggering transcription factors that activate the ER-UPR. A range of secreted anti-microbial peptides, including C-type lectins, likely play a role in effector mechanisms used by *C. elegans* to attack extracellular pathogens, although these mechanisms are still being elucidated. Notably, secreted C-type lectins are also important in mammalian intestinal immunity (Tran and Luallen, 2024). Defense against *P. aeruginosa* may also involve other secreted proteins including lysozymes, proteins showing homology to *Stichodactyla* toxins and CUB-related genes, the expression of which is controlled by the PMK-1/p38 MAPK pathway (Troemel et al., 2006). The ER-UPR is a response conserved from yeast to humans that promotes protein folding and reduces protein synthesis in order to restore homeostasis (Table 1). In addition to this transcriptional response, there is the conserved ER-associated degradation (ERAD) response, a process whereby misfolded proteins in the ER are retrotranslocated into the cytosol for degradation by the proteasome (Lee, 2021). More recently, an ER-associated RNA silencing (ERAS) pathway has been discovered in *C. elegans* and shown to also occur in mammals. The ERAS pathway engages the cell's antiviral RNA silencing system to promote RNA turnover (Efstathiou et al., 2022).

One of the key ER-UPR transcription factors is XBP-1, which activates the ER-UPR when *C. elegans* is growing on *P. aeruginosa* or exposed to bacterial pore-forming toxins (Bischof et al., 2008; Richardson et al., 2010). The ER-UPR activation is PMK-1 dependent, presumably because PMK-1 controls pathogen-mediated upregulation of many secreted factors mentioned above. Interestingly, XBP-1 is essential for larvae to develop when growing on a lawn of *P. aeruginosa* but only when PMK-1 is functional, indicating that, in this case, the immune response can be somewhat harmful (Fig. 4B) (Richardson et al., 2010). In the case of larval arrest when *xbp-1* mutants are grown on *P. aeruginosa*, a mutation in the forkhead transcription factor *fkh-9* allows *xbp-1* mutants to develop at a pace more similar to that of wild-type larvae (Tillman et al., 2018). This benefit was attributed to compensatory activation of ERAD (Fig. 4B). However, activation of ERAD appeared to come at the cost of less proteasomal degradation of cytosolic substrates. This finding provides another example of the balance between proteostasis pathways whereby activation of one pathway can compensate for loss of another.

Blockade of the proteasome can lead to compensatory upregulation of proteasome subunits as controlled by the *skn-1* gene, which encodes multiple isoforms in *C. elegans*. Unlike other SKN-1 isoforms, the SKN-1A isoform has a transmembrane domain at its N-terminus that tethers it to the ER (Fig. 4B), and under basal conditions it is constitutively degraded by proteasomes via the ERAD pathway mentioned above. When the proteasome is blocked, SKN-1A is processed by the peptide N-glycanase PNG-1 and can transit to the nucleus, where it is processed by the aspartic protease DDI-1 to activate gene expression (Lehrbach et al., 2019; Lehrbach and Ruvkun, 2016). This overall process is similar to its ortholog in mammals, NRF1, which is also known to be ER associated.

In a forward genetic screen for *C. elegans* mutants with activation of *chil-27p::GFP* used as a read-out of the oomycete recognition response (ORR), mutants in *png-1* and *ddi-1* were identified to show constitutive reporter expression in the absence of infection (Grover et al., 2024). Further transcriptional analysis found upregulation of many genes in the ORR and the IPR, including all genes that are in common between these two transcriptional defense programs (Fig. 4B). These findings are attributed to build-up of proteotoxic stress as *skn-1a* mutants go through post-embryonic development, due to their defect in upregulating proteasome subunits when needed (Lehrbach and Ruvkun, 2016). Likely due to mounting the ORR and the IPR, *skn-1a* mutants show increased resistance to oomycetes and to *N. parisii* infection. Here, SKN-1A appears to act in a cell-intrinsic manner to repress the IPR and ORR immune responses, acting in the epidermis to regulate oomycete resistance and in the intestine to regulate *N. parisii* resistance (Grover et al., 2024). Interestingly, animals with a hyperactive proteasome have reduced ORR upon exposure to an oomycete trigger, suggesting that induction of the ORR may require stabilization of a factor that is degraded by the proteasome at baseline (Grover et al., 2024). Future studies are needed to investigate the nature of this factor and whether a similarly degraded factor is stabilized upon infection to activate the IPR.

In *C. elegans*, the *skn-1* gene also encodes a protein isoform called SKN-1C that is very similar to SKN-1A except that it lacks the transmembrane domain found in SKN-1A, and thus SKN-1C resides in the cytosol and is not degraded through ERAD (Fig. 4B). In mammals, the SKN-1C ortholog is encoded by a separate gene called *Nrf2*, and SKN-1C/NRF2 have a well-recognized role in activating defense through the oxidative stress response in *C. elegans* and in mammals (Inoue et al., 2005; Attri and Kuwar, 2025; Blackwell et al., 2015). In *C. elegans*, loss of SKN-1C causes susceptibility to infection by bacterial pathogens *E. faecalis* and *P. aeruginosa* (van der Hoeven et al., 2011). Here, ROS induce the p38 MAPK pathway, which acts through SKN-1C to trigger expression of defense genes (Fig. 4B). In a screen for factors required to induce this SKN-1C response, it was found that CDC-48, an ATPase that plays a role in ERAD, was important for the SKN-1C response. Further analyses indicated that loss of CDC-48 did not act through SKN-1A to inhibit the response to bacterial infection but rather activated the ER-UPR also through IRE-1, thereby antagonizing its role in the SKN-1C response (Gabaldón et al., 2024) (Fig. 4B). These findings again highlight the balancing act between proteostasis and immune signaling pathways.

Many of the connections between immunity and the ER in *C. elegans* mentioned above have established connections in humans, or intriguing potential for discovery. For example, the finding that the ER-UPR plays a critical role in promoting *C. elegans* development upon infection (Richardson et al., 2010) was an early discovery that highlighted the critical role of this pathway in handling secreted proteins during infection to promote organismal health. Notably, the ER-UPR is known to be important for immune and inflammatory responses in human epithelial cells. For example, the ER-UPR appears to be dysfunctional in intestinal cells in some cases of inflammatory bowel diseases, with genetic mutations in unfolded protein response (UPR) regulators, including XBP-1, contributing to ulcerative colitis (Hetz et al., 2024). There are also hallmarks of ER-UPR activation in primate cells upon infection with severe acute respiratory syndrome coronavirus 2 (SARS-CoV-2), with increased expression of IRE-1α (Bartolini et al., 2022). In terms of SKN-1C, which studies in *C. elegans* show promotes resistance to infection downstream of ROS generation, there appears to be a key role for its human homolog NRF2 in regulating immune responses in keratinocytes in the context of ROS generation in response to environmental insults to the skin (Salman et al., 2025). It would be interesting to determine whether loss of CDC-48 and subsequent activation of ER-UPR would block NRF2 in human epithelial cells, similarly to effects in *C. elegans*, given that all of the genes have direct orthologs. Finally, it would be interesting to determine whether the build-up of proteotoxic stress in SKN-1A mutants in *C. elegans* that promotes resistance to natural pathogens might also have similar consequences in humans. Human mutations in proteasome subunits that lead to increased IFN-I signaling may have evolutionarily been selected for causing an immune benefit in the context of infection, although the mechanisms and impacts of these mutations are not well understood (Torrelo, 2017; Ebstein et al., 2019; Papa et al., 2020; Zhang et al., 2024).

### Pathways associated with mitochondria

In addition to UPR in the cytosol and ER, an unfolded protein response in mitochondria (mitoUPR) also has connections with immunity, which we cover in this section. The mechanism of mitoUPR activation was discovered in *C. elegans*. Specifically, the mitoUPR is regulated by the transcription factor ATFS-1, which harbors both a mitochondrial-targeting sequence (MTS) and a weak nuclear localization sequence (NLS) (Fig. 4C). Under normal conditions the MTS predominates, and ATFS-1 enters mitochondria, where it is degraded. When mitochondrial stress occurs, mitochondrial import is blocked, and ATFS-1 enters the nucleus, where it induces expression of genes encoding chaperones, such as *hsp-6* and *hsp-60*, which can aid in protein folding in mitochondria (Fig. 4C) (Haynes et al., 2007; Nargund et al., 2012). Among the ATFS-1-regulated genes, there are C-type lectins and other innate immune genes induced by *P. aeruginosa* infection (Pellegrino et al., 2014). Indeed, it was discovered that ATFS-1 localizes to the nucleus upon *P. aeruginosa* infection, inducing expression of C-type lectins and promoting increased survival upon infection.

Another example of the intersection between immunity and mitochondrial proteostasis pathways in *C. elegans* is pathogen-derived small-molecule toxins that target mitochondria and trigger a host response. For example, *P. aeruginosa* produces compounds such as phenazines that disturb the redox state of cells to interfere with mitochondrial function, and compounds such as pyoverdines, which can act as iron siderophores to scavenge iron from the host and cause mitochondrial damage (Hajdú et al., 2024). In liquid growth conditions, *P. aeruginosa*-derived pyoverdine enters *C. elegans*, where it causes mitochondrial collapse and kills the host (Kirienko et al., 2013, 2015; Kang et al., 2018). Targeting the damaged mitochondria for clearance with the autophagy pathway, so-called mitophagy, provides protection against killing by this pathogen. Pyoverdine delivered by *P. aeruginosa* activates the ethanol and stress response element (ESRE) network in *C. elegans*, named for an 11-nucleotide motif found in the promoters of genes induced by stresses including ethanol and ROS (Fig. 4C) (Tjahjono and Kirienko, 2017; Tjahjono et al., 2020). In *C. elegans*, the ESRE appears to be controlled by bZIP transcription factors, including the ZIP-2/CEBP-2 factors mentioned above that control response to translational block, as well as the bZIP transcription factors ZIP-4 and CEBP-1. Furthermore, in keeping with the common theme of antagonism among different proteostasis pathways, there appears to be a stronger ESRE response when the mitoUPR is compromised (Tjahjono et al., 2020). In addition to ESRE, the bZIP transcription factor ZIP-3 also inhibits ATFS-1 in the context of phenazine-associated *P. aeruginosa* infections (Fig. 4C) (Deng et al., 2019).

Thus, there are many mitochondria-related proteostasis pathways that regulate resistance against *P. aeruginosa* infection, perhaps due to the large armamentarium of virulence factors from this broad host-range pathogen that harm mitochondria and its rapid attack on *C. elegans* under certain conditions. Interestingly, the transcription factor that controls the mammalian mitoUPR was discovered to be the bZIP transcription factor ATF5, through functional complementation of *atfs-1* mutants in *C. elegans*, followed by genetic studies in mammalian cells (Fiorese et al., 2016). Subsequently, ATF5 has been shown to promote barrier function during intestinal infection in mice (Chamseddine et al., 2022). ATF5 and the mitoUPR have also been shown to be cardioprotective in mice (Wang et al., 2019), providing an excellent example of how basic findings about proteostasis and immunity in *C. elegans* have been translated into mammals. Similarly, the ESRE network discovered in *C. elegans* is conserved in humans (Kirienko and Fay, 2010), in whom it also responds to ROS and mitochondrial damage (Tjahjono et al., 2020). Notably, in mammals, it has been shown that ROS themselves can induce the mitoUPR, separately from mitochondrial import stress (Sutandy et al., 2023). Findings from the mitoUPR and ESRE are just a few of many examples whereby mitochondria have a role in regulating innate immunity not just in *C. elegans* but also in many other hosts as well. Other roles for mitochondria include serving as an innate immune signaling platform for cytosolic sensors of foreign nucleic acid and releasing mitochondrial nucleic acids that can trigger those and other immune pathways (Kim et al., 2024). Further exploration of how proteostasis in this organelle regulates immune responses represents a rich area for further exploration.

## Future directions: when, where and how?

A recurring theme throughout this Review is the inhibitory relationship among proteostasis pathways under normal conditions. Specifically, there are often compensatory effects whereby inhibition of one pathway will trigger activation of another, reminiscent of hormesis (the phenomenon in which there is benefit from a low-dose treatment of something that causes harm in a high dose). These findings highlight a strategy of organismal resilience to cope with a diverse array of toxins and other insults delivered by pathogens. For example, loss of ERAD appears to trigger the ER-UPR (Gabaldón et al., 2024), loss of SKN-1A triggers the ORR and the IPR (Grover et al., 2024), loss of the mitoUPR increases activation of ESRE (Tjahjono et al., 2020), and loss of the HSR triggers the IPR, ER-UPR and SKN-1A pathways (Kovács et al., 2024). In order to better understand direct effects versus compensatory responses, it is particularly important to perform kinetic analysis after loss of an immune/proteostasis factor. Although application of drugs can provide kinetic information, drug delivery can be challenging in *C. elegans*, owing to its relatively impermeable cuticle; oftentimes, drugs need to be applied at much higher concentrations than in mammalian systems, although there are treatments that will improve cuticle permeability (Partridge et al., 2008; Reinke et al., 2017). Fortunately, the *C. elegans* field has developed several versions of auxin-induced degradation systems, which enable acute depletion of proteins of interest, and that are being used to perform temporal studies (Phanindhar and Mishra, 2023), including those that investigate inherited effects (Willis et al., 2021).

In addition to investigating more precise kinetics of protective responses, there is a need to better understand which tissues are relevant for proteostasis and/or immunity in different contexts. All of the findings described in this Review focused on intracellular proteostasis, including a clear role for cell-intrinsic responses in some cases (Grover et al., 2024). However, there also appears to be an intriguing role for extracellular proteostasis in defense against pathogens (Gallotta et al., 2020). Furthermore, there is a rich and growing literature of findings in *C. elegans* focused on systemic proteostasis and immunity (Hoffman and Aballay, 2019; Wani et al., 2020; Bar-Ziv et al., 2020a; Hodge et al., 2022; Miles et al., 2019). Here, the simple body plan of *C. elegans* provides a convenient model to understand coordination among different tissues. Because these findings have not directly explored immunity and have been reviewed several times, they are not covered in this Review, but are a rich area for investigation in mammalian systems, in which they are less well understood (Hetz et al., 2024). These many examples of systemic signaling in *C. elegans* emphasize the importance of incorporating tissue-specific analyses, which are available via the auxin-degradation system mentioned above, as well as through tissue-specific RNA interference and through traditional tissue-specific rescue. Ideally, these tools will be combined with more studies that compare pathogen load versus disease tolerance metrics as well as kinetics of infection over time, to better understand the mechanisms by which proteostasis pathways intersect with immunity. For example, it is important to know when pathogen entry or replication is blocked, and when pathogens themselves have been cleared, which has rarely been described, but can be illuminated with kinetic studies (Balla et al., 2015; Castiglioni et al., 2024; Balla et al., 2019). Also of interest is how immunity and proteostasis change over the lifespan, with recent findings indicating that some factors, such as ZIP-2, have different roles during aging (Hahm et al., 2020; Taouktsi et al., 2023).

In terms of relevance to mammals, we have noted above when findings on immunity and proteostasis discovered in *C. elegans* are conserved in mammals, such as the roles of the ER-UPR (Hetz et al., 2024), TFEB/autophagy (Kuo et al., 2018; Brady et al., 2018) and the mitoUPR (Zhou et al., 2022). Furthermore, we have made suggestions for findings from *C. elegans* that could be further explored in mammals, such as overlapping uORFs in CEBP transcription factors in possibly sensing pathogen blockade of translation elongation. Another area for exploration is the age-related impacts on insulin-signaling and HSF-1 to control bacterial resistance, or age-related impacts on proteasomal degradation of RIG-I to control viral resistance. One takeaway from this Review is the surprising benefits that occur in *C. elegans* upon proteasomal dysfunction, including increased immunity against epidermal and intestinal pathogens, as well as tissue-specific reduction in protein aggregates. These effects relate to the IPR and ORR programs, which are still being defined, but can be triggered by the proteasome inhibitor bortezomib. Interestingly, bortezomib is a drug used in the clinic (Velcade) for treating multiple myeloma with a complex mechanism of action still being debated, including inhibition of NFκB signaling (Sogbein et al., 2024), which is lacking in *C. elegans* (Irazoqui et al., 2010b). Perhaps there is a human equivalent of the IPR and ORR that has a role in the therapeutic effects of bortezomib in humans. We also note the many human variants in proteasome subunits that are associated with increased IFN-I signaling through poorly understood mechanisms (Goetzke et al., 2021). Overall, these investigations will help us better understand connections between proteostasis and immunity, as well as how this relationship regulates aging-associated diseases and other aspects of organismal health.
